# Harnessing Time‐Dependent Magnetic Texture Dynamics via Spin‐Orbit Torque for Physics‐Enhanced Neuromorphic Computing

**DOI:** 10.1002/advs.202513946

**Published:** 2025-11-10

**Authors:** Yifan Zhang, Yu Li, Huai Lin, Xinying Wang, Long Liu, Guoliang Xing, Di Wang, Zhihao Zhao, Zhipeng Guo, Junbo Yang, Jiebin Niu, Yan Sun, Tom Wu, Guozhong Xing

**Affiliations:** ^1^ State Key Laboratory of Fabrication Technologies for Integrated Circuits Institute of Microelectronics Chinese Academy of Sciences Beijing 100029 China; ^2^ University of Chinese Academy of Sciences Beijing 100049 China; ^3^ VeriSilicon Technology (Shanghai) Co., Ltd. Shanghai 201306 China; ^4^ School of Civil Engineering and Architecture Northeast Electric Power University Jilin 132012 China; ^5^ Jilin Special Equipment Inspection and Research Institute Jilin 132013 China; ^6^ Shenyang National Laboratory for Materials Science Institute of Metal Research Chinese Academy of Sciences Shenyang 110016 China; ^7^ Department of Applied Physics The Hong Kong Polytechnic University Hong Kong 999077 China

**Keywords:** advanced neuromorphic hardware, combinatorial optimization problems, domain wall motion, magnetic texture, pattern recognition, spin‐orbit torque

## Abstract

Performing visual recognition and combinatorial optimization simultaneously on a single multifunctional neuromorphic computing platform offers significant advantages in terms of efficiency, real‐time processing, and integrated decision‐making. However, the advances are hindered by hardware constraints. Here, a new type of all‐electrically controlled labyrinth magnetic texture (MT) devices is reported, wherein the trainable MT can be created, manipulated, and detected efficiently and reliably at room temperature. By utilizing the spin‐orbit torque (SOT) effect, it can modulate the nonlinear magneto‐resistance of the device via a corroborated dynamic conductance matrix, mimicking the mixed short‐term and long‐term potentiation of biological synapses. The developed SOT‐MT devices excel in diverse in‐memory computing tasks, including pattern recognition and combinatorial optimization. Utilizing a cross‐bar array with single SOT‐MT devices, outstanding test accuracy is achieved over 93% on MNIST, and a success rate exceeding 95% in solving the 8‐city traveling salesman problem with the Hopfield network. Synergistic tailoring of constant and dynamic fluctuations contributes to this success. The study paves the way for dynamic network MT devices, advancing complex task processing by enabling efficient fusion of cognition and combinatorial optimization on a single neuromorphic hardware system.

## Graphical abstract

A neuromorphic computing platform using spin‐orbit torque‐controlled magnetic textures is reported. The device implements bio‐inspired synaptic functions and achieves high performance in both pattern recognition (>93%) and combinatorial optimization (>95%), enabling unified processing of cognitive and optimization tasks.

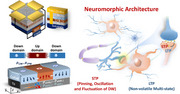

## 1 Introduction

Neuromorphic hardware, as building blocks of an alternative computing paradigm to traditional digital circuits confronting the von Neumann bottleneck and the stagnation of Moore's Law, has emerged as a forefront of cutting‐edge research in the last few years.^[^ ^1^, ^2^, ^3^, ^4^ ^]^ Emulated by the brain's structure and functionality, neuromorphic hardware enables efficient brain‐inspired computations with key features such as massive parallelism and ultra‐low power consumption. The term “neuromorphic” was coined by Carver Mead in the 1990s to describe very large‐scale integration computing systems that combine analog and digital signals.^[^ ^5^ ^]^ In recent years, the demand for low‐power computing, the end of Dennard scaling, and the rise of deep learning have reinforced the importance of neuromorphic hardware.^[^ ^6^, ^7^, ^8^ ^]^

**Figure 1:**
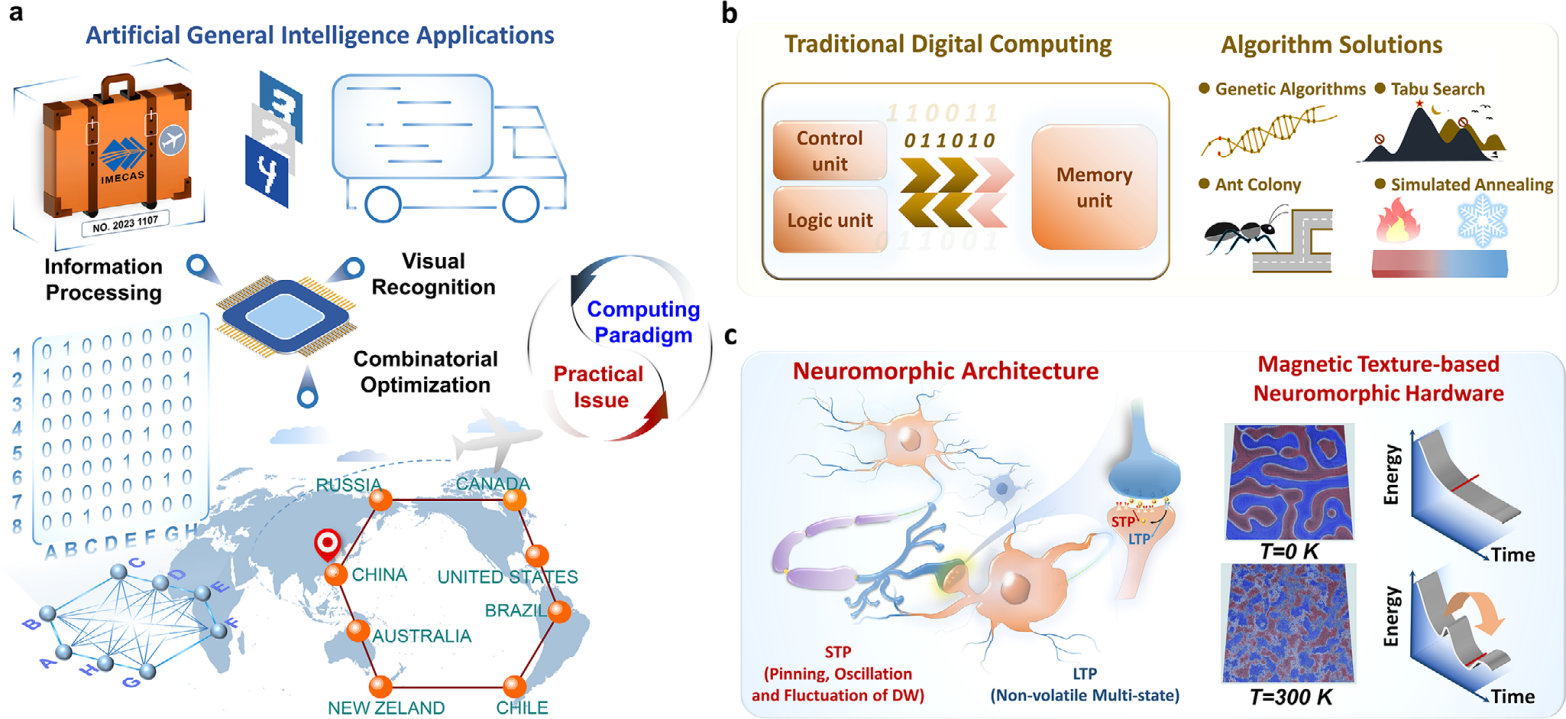
a) Exemplified practical issue and implementation of emerging computing paradigm for sustainable advanced computing platforms. b) Traditional computing architecture, methods, and algorithm models. c) Inherent fluctuations and advantages in identification and combinatorial optimization leveraged by MT‐based neuromorphic hardware.

As demands for complex cognitive tasks and optimization becomes more prevalent and refined, applications such as logistics and *e*‐commerce, autonomous vehicles, and industrial automation are faced with demanding requirements for real‐time recognition^[^ ^9^, ^10^ ^]^ and combinatorial optimization.^[^ ^2^, ^6^ ^]^ To address these challenges in real‐life applications, there is a pressing need for integrated decision making and efficient operations by combining visual recognition with optimization algorithms, as depicted in **Figure** **1**a. While significant progress has been made in traditional digital computing schemes and algorithmic learning techniques (Figure 1b), there remain challenges related to hardware intelligence, efficacy constraints, synergistic training, and scalability that need to be tackled.^[^ ^10^ ^]^

Developing neuromorphic hardware systems involves various architectures and devices, including floating gate, memristors, spintronics, and combining analog and digital approaches.^[^ ^11^ ^]^ Among these, the unique topological morphology of labyrinthine magnetic texture (MT) holds significant potential with high‐dimensional dynamic responses for information storage and time‐ and space‐domain neuromorphic computing. Nevertheless, information carriers such as single‐domain, skyrmion, and labyrinthine domain devices present compelling prospects for analog operations, low power consumption, and compact form factors.^[^ ^12^, ^13^ ^]^ Particularly, labyrinthine MTs, with their complex and dynamic behaviors, offer a promising solution as transition states in generating skyrmions or magnetic bubbles. The development of brain‐like synapses based on labyrinthine domains opens up possibilities for implementing Hebbian learning rules and realizing sophisticated neuromorphic computing.^[^ ^14^, ^15^, ^16^ ^]^ However, challenges persist in maintaining stability, controlling dynamic MTs, and achieving all‐electric field manipulation at room temperature.

In this study, we report an advancement of multifunctional neuromorphic hardware implementation with spin‐orbit torque magnetic textures (SOT‐MTs) that remain stable at room temperature. By all‐electrically controlling the MT through SOT, we achieve 16 distinguishable resistance states in the device. The magnetoresistance (MR) response of devices can be modulated by the dynamic MT evolution with trainable magnetic domain wall (DW) motion, enabling the manipulation of synaptic weights in neural network calculations. We demonstrate the applicability of our approach by performing recognition tasks on the modified National Institute of Standards and Technology (MNIST) handwritten dataset and multi‐task recognition of the traveling salesman problem (TSP). The incorporation of dynamic fluctuations improves the success rate of optimal solution searches, as depicted in Figure 1c, corroborating the potential applications of MT device hardware in advanced neuromorphic computing.

## 2 Result

### 2.1 Dynamic Nonlinear Magnetoresistance Response in Complex Magnetic Texture

**Figure 2:**
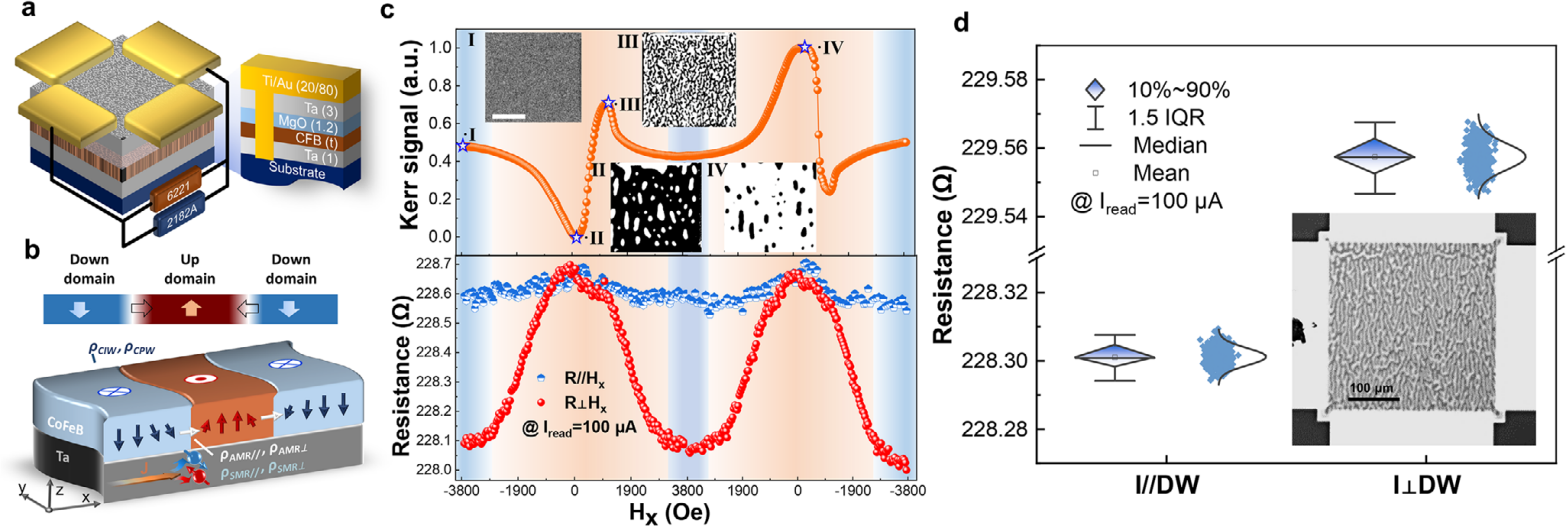
a,b) Schematic of the device and 3D structure of periodic MT and the MR‐related physical parameters. c) MR vs in‐plane current in *x* and *y* direction, and MOKE images with Kerr signals under H_x_ sweeping. d) Resistance of the MT device with read current in perpendicular and parallel orientations to the domain alignment. The inset illustrates the aligned MTs.

A series of Ta(1)/Co_20_Fe_60_B_20_(*t*)/MgO(1.2)/Ta(3) [(*t* = 1.1–1.5 nm)] multilayers on Si/SiO_2_ substrates as schematically illustrated in **Figure** **2**a were grown by magnetron sputtering. The films' stack system with a structure of t = 1.3 nm was examined using complementary bright‐field scanning transmission electron microscopy and energy dispersive spectrometer measurements. The analysis of the results revealed a flat interface with a high‐quality, atomic‐level expected films stack structure (refer to Figure S1a,b, Supporting Information). As the thickness (*t*) of CoFeB increases, the perpendicular magnetic anisotropy (PMA) of the film stack gradually weakens until *t* reaches 1.4 nm. Within the range of *t* = 1.1–1.3 nm, a complex MT with a random distribution is observed to stably exist at room temperature, even without the presence of an external magnetic field. Additionally, the density of magnetic DW increases with the thickness of CoFeB (Figure S2, Supporting Information).

To gain insights into the effect of MT evolution on the device MR, we quantitatively characterized the influence of complex MTs on the overall resistance. After the complex magnetic DWs are randomly formed, an external magnetic field is applied to induce the device to form an orientation‐arranged parallel magnetic domain structure. The dependence of MR on the MTs can be expressed as:^[^ ^17^ ^]^

(1) $$\begin{array}{ccc} R_{⊥}-\;R_{//} & = & \frac{\delta_{w}}{d}\;\left(R_{CPW}-R_{CIW}\right)+\left({R_{AMR}}_{⊥}+{R_{SMR}}_{⊥}\right)\\ & & -\left({R_{AMR}}_{//}+{R_{SMR}}_{//}\right) \end{array}$$

where *R_//_* and *R_⊥_* are the resistance values measured when the magnetic DWs are arranged in parallel and the read current is parallel and perpendicular to the dominantly arranged magnetic DWs, respectively. *δ*_W_ refers to the width of magnetic DWs, and *d* is the size of magnetic domains. Both *δ*_W_ and *d* are related to the exchange anisotropy constant, Dzyaloshinskii‐Moriya Interaction (DMI) coefficient (see Figure S3, Supporting Information for more information).^[^ ^18^ ^]^ *R_CIW_* and *R_CPW_* are the resistances parallel and perpendicular to the current caused by the presence of magnetic DWs, respectively. *R_AMR//_*, *R_AMR⊥_* are the anisotropy MR (AMR) changes caused by the angle between the magnetization direction and the current, respectively; and *R_SMR//_* (*R_SMR⊥_*) is the contributions of the spin Hall magnetoresistance between the interfaces induced by spin‐orbit interaction when the current is parallel (perpendicular) to the magnetic domain orientation.^[^ ^17^, ^19^ ^]^

The 3D image of the device and domain structure (Figure 2b) displays the individual magnetoresistive components. The magneto‐optic Kerr effect (MOKE) images in the inset of Figure 2c elaborate the dynamic evolution of MT. At the saturation field, the magnetization tends to in‐plane saturation, resulting in minimal MR. As the magnetic field gradually decreases to zero, the device spontaneously forms a periodic magnetic domain structure. This controllable deformation and evolution of MTs, driven by dynamic process stimuli, reliably modulate the overall magnetoresistive response of the device. This experimental observation supports the realization of multifunctional neuromorphic hardware at both an empirical and a mechanistic level. Figure 2d illustrates the resistance of the current perpendicular to the parallel orientation of magnetic domains, and the calculated value (*R*_⊥_‐*R*_//_)/*R*_s_×100% is 0.28%.Additionally, a measurement of (*R_AMR⊥_ + R_SMR⊥_ – R_AMR//_ – R_SMR//_*)/*R_s_* ×100% = 0.12% was obtained from Figure 2c, when an in‐plane saturation magnetic field was applied, revealing the dynamic evolution of MT as the MR changes. Consequently, the calculation $\frac{\delta_{w}}{d}$ (*R_CPW –_ R_CIW_)*/*R_s_* ×100% resulted in a value of 0.16% with a nontrivial change of resistance, wherein the parametric indexes are comparable to the reported anisotropy of domain wall resistance.^[^ ^17^ ^]^

### 2.2 Dynamic Magnetic Texture Characteristics Driven by SOT

**Figure 3:**
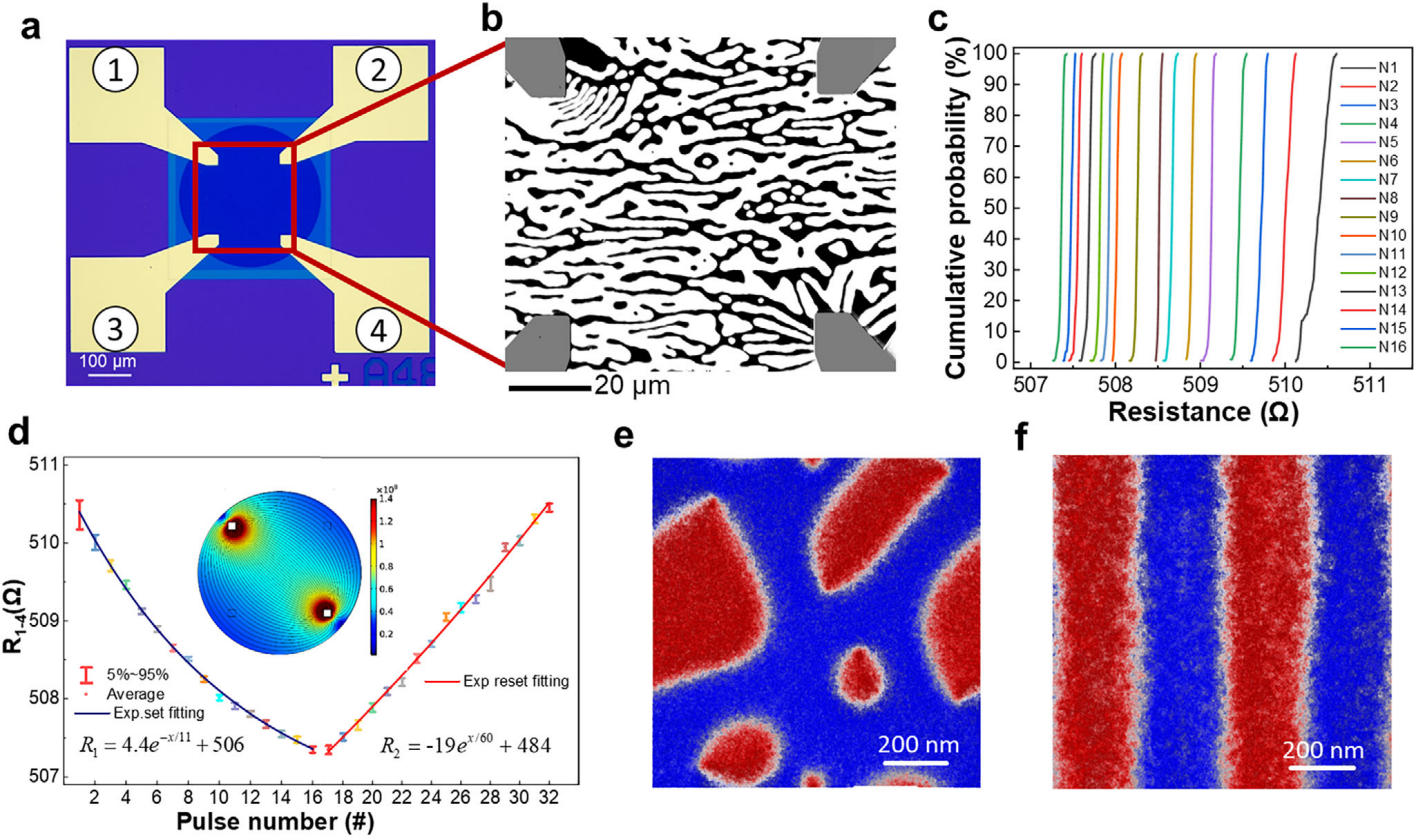
a) Optical micrograph of single MT memristor and b) corresponding labyrinth DW MOKE images driven by current pulses. c) Cumulative probability distribution of 16‐state and 200‐point for each state. d) Measured changes in synaptic weight vs applied pulse numbers under set and reset. Boxplots show the fluctuations in reading processes. Inset depicts the reading current density in Ta layer. e) Random domain distribution in the initial state simulated by MuMax^3^. f Parallel MT generated upon current driving.

By harnessing the underlying mechanism of MR/conductance matrix alteration in our developed devices, our objective is to utilize Ta/CoFeB MT devices to achieve synapse weight modulation that closely resembles the synaptic plasticity observed in the human brain. This modulation will be accomplished by applying electric pulses. In our experimental setup, a single device (**Figure** **3**a) was utilized as an artificial synapse at room temperature, without the need for an external magnetic field. Activation involved applying a 500 µs current pulse of 35 mA between node 1 and node 4, inducing the movement of the magnetic DW. Varying current density between the input and output terminals resulted in distinct textural characteristics^[^ ^20^ ^]^ (Figure 3b). The distribution of resistance values for each state, shown in Figure 3c, demonstrated 16 nonoverlapping resistance states that enable precise and differentiated setting of synaptic weights. Conversely, in the inhibitory state, applying negative pulses (I = −5 mA, *t*_p_ = 500 µs) between node 1 and node 4 increased the resistance *R*_1‐4_, functioning as an inhibitor (Figure 3d). Due to the non‐alignment of lateral inhibitory pathways between node 2 and node 3, higher reset current density and pulse width were required. While the experimental pulse durations (0.5–5 ms) in our large‐scale (≈100 µm for MOKE probing dynamics) device exceed typical electronic requirements, they were necessary to resolve complex domain wall dynamics under current experimental constraints. Crucially, scalability simulations based on experimentally derived parameters (Table S1, Supporting Information) confirm that the underlying principle holds at reduced dimensions. At a projected 1 µm × 1 µm scale, identical current density drives analogous magnetic ordering perpendicular to current flow within 500 ps. This demonstrates the pathway toward sub‐microsecond operation competitive with state‐of‐the‐art neuromorphic implementations upon device miniaturization.

To reveal the formation state and dynamic process of complex magnetic DWs, we characterized the basic magnetic parameters of the device (see Figures S2 and S3, Supporting Information), and used MuMax^3^ micromagnetic simulation^[^ ^21^ ^]^ to show that the device spontaneously formed Nèel‐type MT without an external field (see Table S1, Supporting Information for parameters). Based on forming and stabilizing magnetic domains, we systematically developed all‐electrically controlled SOT‐MT devices. The random distribution depicted in Figure 3e is formed through simulations conducted under certain initial conditions. The resulting distribution exhibits a mixed structure of stripes and magnetic bubbles, which is comparable to the magnetic domain distribution observed under zero field, as shown in Figure 2c via MOKE characterizations. The presence of through‐holes in the device gives rise to a mixed driving mode of Spin Transfer Torque (STT) and SOT associated with the current that drives the device. Under the induction of 16 current pulses (*J*_e_ = 2.15 × 10^11^ A m^−2^, *t*_P_ = 0.5 ns), the magnetic domains undergo a transformative process, aligning in a parallel configuration, as illustrated in Figure 3f. Subsequently, when a read voltage is applied to the device, the current distribution within the device undergoes modifications due to the presence of the magnetic DW. This alteration is experimentally observed as an MR change of the device, as presented in Video S1 and Figure S4 (Supporting Information).

To investigate the impact of STT and SOT, we employed a decoupling approach and utilized MuMax^3^ for independent analysis. In our study, we applied a hybrid STT/SOT current density to the device, which was consistent with experimental values. The application of this hybrid current density resulted in a transformation of the MT from an initial random morphology to a parallel orientation, mirroring the observations made in experiments (Video S1, Supporting Information). On the other hand, when only STT was employed, the overall morphology of magnetic domains remained random, with minimal changes in response to STT current pulses (Video S2, Supporting Information). In contrast, the utilization of SOT pulses to drive DWs led to a dynamic process where disorderly arrangements transitioned to orderly organizations (Video S3, Supporting Information). These simulation results corroborate the substantial role of SOT in governing the motion of MTs.

### 2.3 Plasticity of SOT‐MT Neuromorphic Devices to Adapt in Response to Stimuli

**Figure 4:**
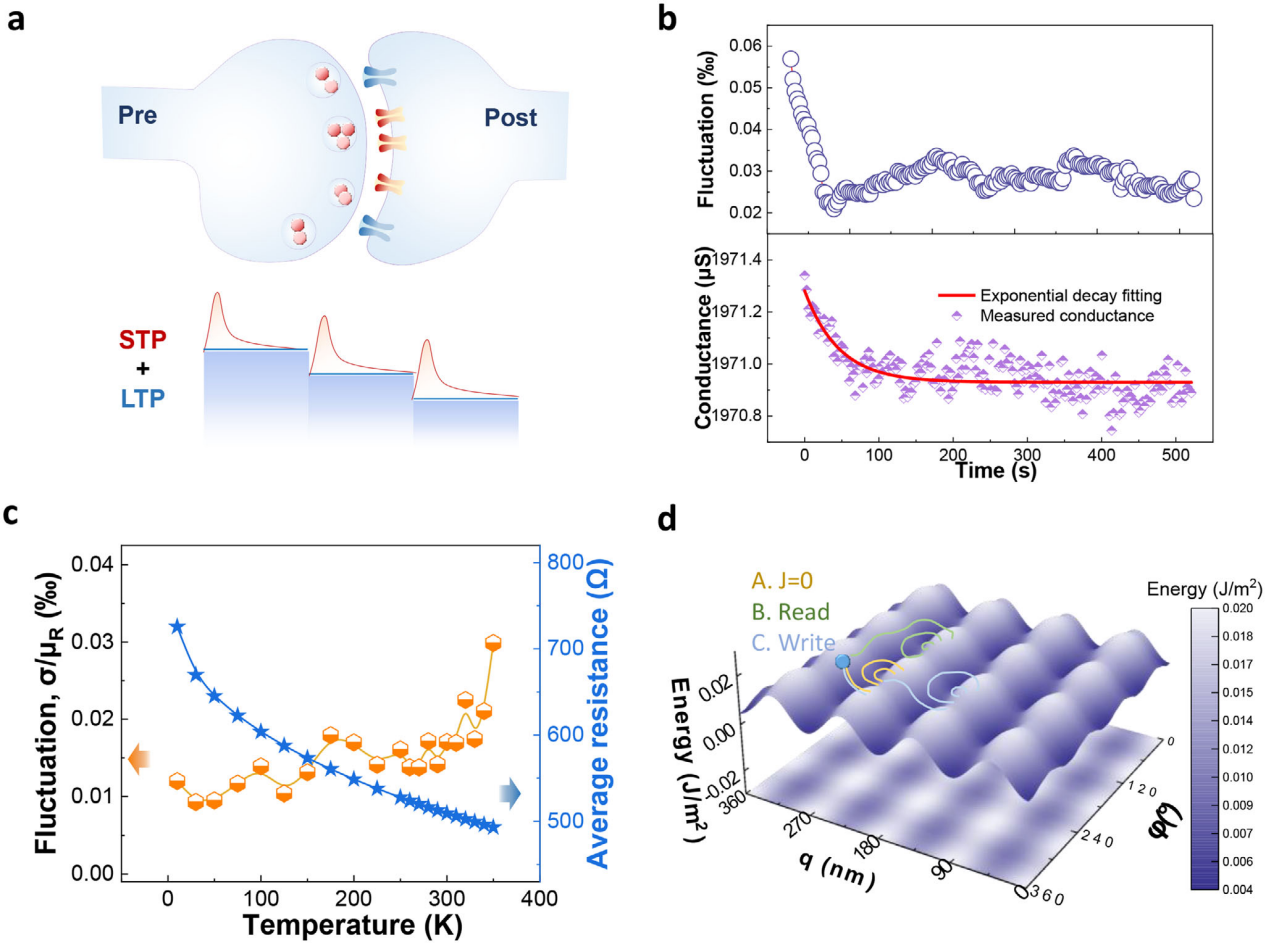
a) Schematic of mixed STP and LTP properties of developed MT synapses. b) Conductance modulated by set pulse (lower panel) and the decay fluctuation (upper panel). c) Device resistance and fluctuation data versus temperature. d) Energy landscape of DW motion in different potential wells under external stimuli.

Following the application of an active driving pulse, we conducted a series of 200 read pulses to examine the time‐dependent behavior of device conductance. Figure 3d illustrates that, in a typical high‐impedance state, the device experiences a rapid decline in conductance from a higher value to a lower value immediately after the current pulse, eventually reaching a stable state over time. The 16 adjustable states of the device exhibit a combination of both long‐term plasticity (LTP) and short‐term plasticity (STP) processes, bio‐logically emulating the real synapses (**Figure** **4**a). This behavior clearly mimics the dual‐timescale (short‐ and long‐term) plasticity inherent to biological synapses, a feature our device reproduces via its tunable magnetoresistive response and intrinsic fluctuation dynamics. The decay of conductance, denoted as Δ*G_D_* (*t*) in Figure 4b, adheres to an exponential relationship: Δ*G_D_* (*t*) =  Δ*G*_0_ *exp*(− *t*/τ), where Δ*G*_0_ represents the instantaneous change in conductance at t = 0 [Δ *G*_0_ =  *G*(*t*  =  0) − *G_i_*], and τ is the attenuation coefficient governing the regulation of STP based on LTP, as shown in Figure 4a.

During the decay process, we observed random fluctuations in the conductance value of the device. The conductance was modeled as *G* (*t*) = *G_i_*  + Δ*G_C_* + Δ*G_D_* (*t*), where *G_i_* is the ideal conductance, Δ*G_C_* is constant Gaussian. The upper panel of Figure 4b shows that the device's fluctuation gradually decreases over time, indicating a characteristic fluctuation attenuation. The fluctuation of each resistance state of the device (*F = σ/µ*) can be controlled between 0.03‰ and 0.23‰, where σ is the standard deviation and µ is the average value of the device conductance (see Figure 3d; Figure S5, Supporting Information) at room temperature. To assess the conductance fluctuation (*F = σ/µ*) of the device, variable temperature measurements were conducted as shown in Figure 4c. At stable temperature points within the range of 10–350 K, 500 readings were taken to examine the fluctuation behaviors. The data revealed an increasing trend in overall fluctuation, with values between 0.01‰ and 0.03‰, even at elevated temperatures. Notably, a fluctuation of 0.015‰ was observed at room temperature, approximately one order of magnitude smaller than the fluctuation attenuation observed in each resistance state of the device. This experimental observation aligns with previous reports in the field. It can be argued that the temperature variation of anisotropy in CoFeB may induce minor changes in the wall thickness; however, the impact is anticipated to be minimal. Additionally, another factor that could influence DW scattering is the spin mixing relaxation time.^[^ ^17^ ^]^ Our results, as depicted in Figure 4, support the notion that the temperature‐dependent scattering ρ_0_^↓(↑)^(*T*) in CoFeB is spin‐independent, leading to a significant reduction in the contribution of DW scattering as the temperature increases.^[^ ^22^, ^23^ ^]^ Additionally, a qualitative energy analysis (Figure 4d) was performed using a simplified *q‐φ* model to represent the aligned magnetic domains and derive the energy distribution.^[^ ^24^, ^25^ ^]^ In the absence of external current excitation, the magnetic domain system undergoes fluctuations within the potential well, leading to a statistically Gaussian distribution of resistance values. When a read current is applied, the magnetic domain acquires energy to traverse the potential barrier and relax into another energy potential well, resulting in fluctuations. However, the read current‐induced energy change is small and does not cause depinning from the external potential barrier, preserving the resistance state of LTP. Under the influence of a large write current excitation, the magnetic domain alignment allows the system's energy to surpass the potential barrier, leading to depinning. This results in a macroscopic resistance state switch, followed by relaxation into the energy potential well.

The observed conductance attenuation and fluctuation can be attributed to factors such as the tilting of the magnetic domain wall,^[^ ^26^ ^]^ postpinning oscillations,^[^ ^27^ ^]^ and temperature perturbations influenced by the magnetic domain itself after current driving. The four‐terminal device described above exhibits characteristics that encompass both the activation and inhibition of LTP. It also adheres to the fundamental Hebbian learning rules observed in human brain learning, as well as the anti‐Hebbian learning rules related to the mutual inhibition of lateral neurons.^[^ ^28^ ^]^ Additionally, during the time‐dependent decay process, it incorporates a combination of STP and LTP when presynaptic neurons release signals to postsynaptic neurons. These features provide a potential solution for enhancing brain‐like neuromorphic devices.^[^ ^29^, ^30^ ^]^

### 2.4 Integration of Complex SOT‐MT Devices and Neural Network Optimization

**Figure 5:**
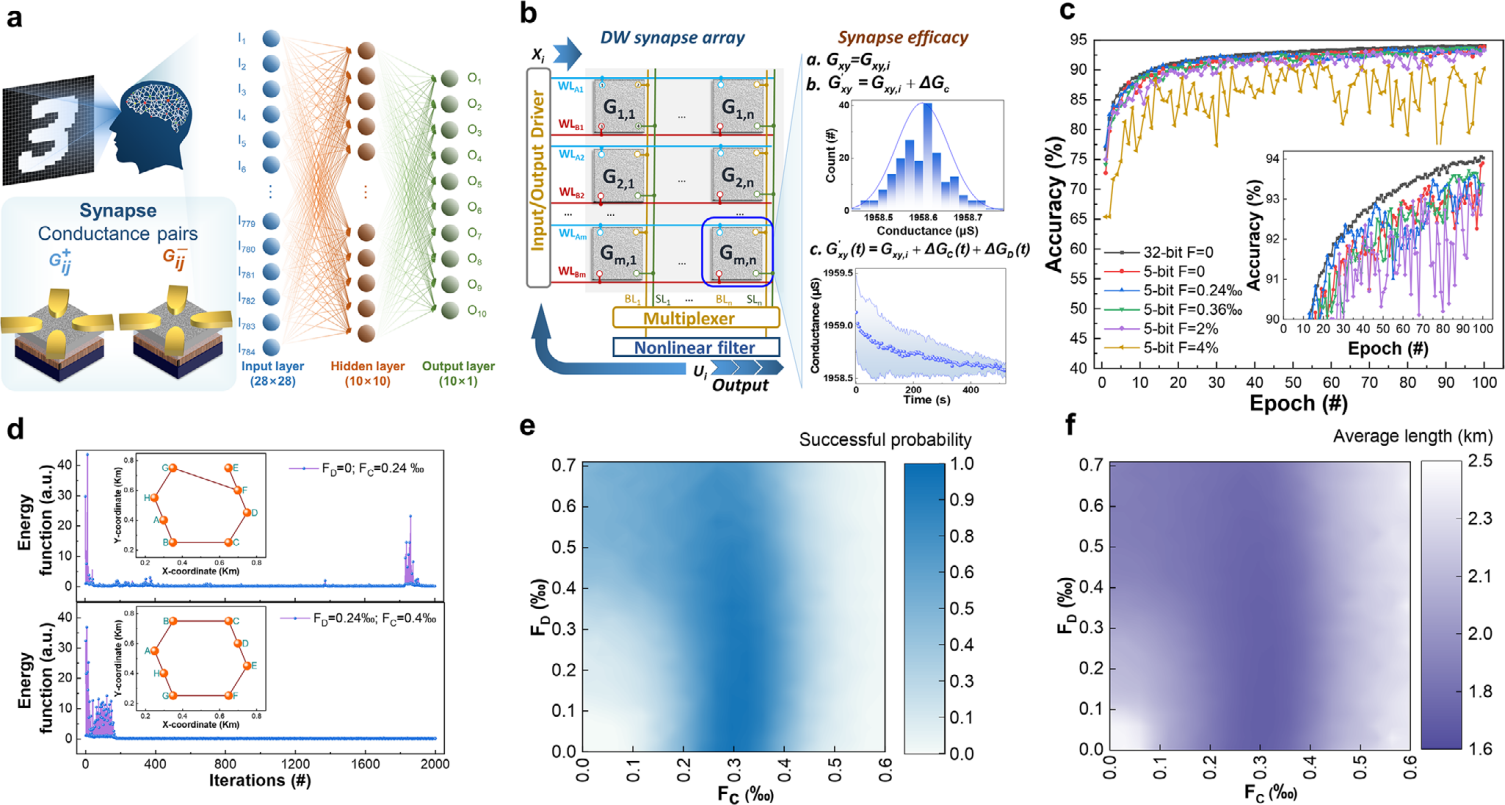
a) Tri‐layer neural network for MNIST pattern recognition. b) Schematic of SOT‐MT devices array. Measured Network weights of 16‐state are mapped into normalized resistance states. c) Accuracy evolution during training for the MNIST task with read noise only, both measured read and write noise. The inset provides a magnified view of the final 80 epochs. d) Fixed Gaussian fluctuation, mixed STP and LTP fluctuation of SOT‐MT synapses for HNN solving 8‐city TSP. Insets: Final optimization paths. Diagrams of e) success probability and f) average trial length of 1000 solutions vs F_c_.

Based on the experimental data from our prototype devices, we co‐designed device arrays to enhance multitasking performance. For the recognition task on the MNIST handwritten dataset,^[^ ^13^, ^31^, ^32^, ^33^, ^34^, ^35^, ^36^, ^37^ ^]^ we used a neural network with a 784‐neuron input layer, 100‐neuron hidden layer, and 10‐neuron output layer. Conductance pairs composed of two devices were utilized as 5‐bit artificial synapses (**Figure** **5**a). This paired‐cell design not only enables differential weight representation but also provides a foundation for implementing differential readout schemes to mitigate common‐mode disturbances such as temperature drift. The performance of our MT devices was compared to software‐based approaches, demonstrating a recognition accuracy close to full precision (94.0%) and outperforming existing spin device‐based networks in terms of energy efficiency and speed, as demonstrated in **Table** **1**. While prior works have utilized topological magnetic states (e.g., skyrmions) as non‐volatile synaptic weights analogous to memristive devices, our work advances the field through three critical innovations. Nevertheless, we exploit the inherent spatiotemporal dynamics of labyrinthine domain walls—including stochastic relaxation and inter‐domain interactions—which remain underexplored in neuromorphic systems. Importantly, our developed all‐electrical SOT control eliminates external magnetic fields, enhancing scalability and CMOS compatibility. Furthermore, leveraging these dynamics algorithmically: time‐dependent conductance fluctuations actively improve recognition accuracy (MNIST >93%) and combinatorial optimization success rates (TSP >95%) in Hopfield networks. This synergistic hardware‐algorithm co‐design transcends conventional weight storage, establishing a pathway for physics‐enhanced neuromorphic computing.

**Table 1:** Comparison of representative memristor device‐based MNIST tasks implementation.

References	Synapse device	External magnetic field	Write speed [ns]	Energy consumption/bit [fJ]	Energy consumption of array [µJ]	Recognition accuracy
[13]	Skyrmion, Pt /GdFeCo/MgO	Yes	0.05	16.7	0.55	1024‐100‐10 Ideal software, 93% Device fluctuation, 89%
[31]	SOT‐DW‐Hall bar, MgO/CoFeB/W/Ta	Yes	10	33.3	1.67	784‐128‐10 93%
[32]	SOT Hall bar, Pt/Co/AlO_x_	Yes	6 × 10^7^	≈8 × 10^11^	3.01 × 10^10^	784‐300‐10 Ideal, ∼95% Fluctuation = 2%, ∼80%
[33]	Random number generators Ta/CoFeB/MgO/Ta	Yes	6.25	466	0.36	784‐50‐10 1024‐bit, 97.23%
[34]	SOT Hall bar, Ta/Pt/CoGd/Ta	Yes	10	≈6.57 × 10^6^	1.13 × 10^5^	784‐100‐10 93%
[35]	SOT Hall bar, MgO/FePt/TiN/NiFe	No	5 × 10^4^	3.46 × 10^7^	3.91 × 10^3^	784‐3‐3 Ideal software, 95.04% Device based, 91.17%
[36]	Ge_2_Sb_2_Te_5_	No	200	Set: 8.8×10^6^ Reset: 6.9×10^6^	1.1 × 10^3^	196‐4‐10 86%
[37]	TiN/TaO_x_/HfO_x_/TiN	No	Set: 50 Reset: 200	Set: 272.58 Reset: 214.41	0.81	784‐100‐10 Off‐chip learning, 94.9% On‐chip learning, 92.3%
Our work	SOT‐MT Ta/CoFeB/MgO/Ta	No	0.5	31.25	0.39	784‐100‐10 Ideal software, 94.06% Fluctuation = 0.36‰, 93.72% Fluctuation = 4％, 92.15%

Regarding software‐hardware co‐implementation, the SOT‐MT devices operate within a crossbar array architecture (Figure 5b) to enable in‐memory computing. During operation, row drivers and multiplexers select specific columns, allowing the summation of read currents along a column to inherently perform multiply‐accumulate (MAC) operations. This summed current is then processed by a nonlinear filter acting as the neuronal activation function, generating the input signal (U_i_) for subsequent layers or cycles, consistent with established neuromorphic approaches.^[^ ^3^ ^]^ For training, the learned algorithmic weights (W_ij_) from software simulations (e.g., for MNIST or HNN) are linearly mapped onto the device conductance (G_ij_) via W_ij_ = a·G_ij_ + b, enabling hardware deployment.^[^ ^38^ ^]^ Implementing this requires integrated peripheral read/write circuitry to accurately program conductance and read out results, as demonstrated in our previously developed magnetic memory systems.^[^ ^39^ ^]^

Figure 5c depicts a comparison of recognition accuracy between software and MT devices, both employing the same network architecture. The trained weights were discretized into 5‐bit values and subsequently mapped onto synaptic arrays. Following this mapping, the device demonstrated a gradual transition to a stable fluctuation state, right after the pulsed current writing with non‐trivial fluctuation/noise data as experimentally derived from Figure 3. The network's performance was evaluated under increasingly realistic device conditions. The parameter *F* represents the fixed Gaussian conductance fluctuation. When considering only these read fluctuations (*F* = 0.24‰, 0.36‰), a final accuracy of 93.72% was achieved. To provide a more rigorous assessment, we incorporated the cycle‐to‐cycle and/or device‐to‐device write variation (up to 4%) observed experimentally in Figure 3d into the weight update process during training. This combined read and write noise model resulted in a final accuracy of 92.15%, demonstrating the network's resilience to the inherent non‐idealities of our SOT‐MT synapses and its performance proximity to the ideal software baseline of 94.06%. When compared to existing neural networks based on spintronic devices, this approach offers enhanced energy efficiency and processing speed, as detailed in Table 1. Importantly, the operation of MT devices does not necessitate an external magnetic field, representing a significant advantage in terms of integration and scalability within system architectures.

In combinatorial optimization problems, the MT devices offer an optimization scheme based on intrinsic characteristics without introducing random sources through peripheral circuits. This approach improves the probability of optimization, particularly in escaping local optimal solutions in the traveling salesman problem.^[^ ^40^ ^]^ By leveraging the magnetic domain fluctuations inherent in the MT devices and considering fluctuations introduced by the pre‐neuron, we achieved the optimal iterative outcome and obtained the optimal path for the 8‐city traveling salesman problem. The MT devices' ability to operate without an external magnetic field simplifies integration, system scaling, and practical implementation.

To maximize the success rate of optimization, we examined the principle of the Hopfield neuron network (HNN) and the fluctuation characteristics of the device to find the best approach. The Gaussian fluctuations and the decay were quantized as *F_C_* =  σ/μ and as *F_D_* =  Δ*G*_0_/μ, where the instantaneous conductance change value Δ *G*_0_ =  *G*(*t*  =  0) − *G_i_*. On this basis, we conducted an analysis of the success rate phase diagram using various fluctuations (Figure 5e). When the device has an ideal conductance value (*F_C_* = 0, *F_D_* = 0), it is unable to escape local minimums, resulting in an optimal success rate close to zero. For a fixed Gaussian fluctuation (*F_C_* ≠0, *F_D_* = 0), smaller fluctuations are unable to reach the global optimum, while larger fluctuations (*F_C_* >0.4‰) cause the energy to escape the lowest value, leading to a decrease in the success rate. This is illustrated by the energy function decline curve^[^ ^40^, ^41^ ^]^ in the upper panel of Figure 5d. Consequently, when optimizing the TSP with fixed Gaussian fluctuations, there is a greater demand for uniformity in the device and the fluctuation window.

On the other hand, when the device exhibits mixed STP and LTP fluctuations (*F_C_* ≠0, *F_D_* ≠0), the lower panel of Figure 5d shows that the MT device gets rid of the local optimal solution and converges to the global optimal during the iterative process. Therefore, the device array realizes the optimization of the 8‐city TSP within the fluctuation range of the device (*F_C_* = 0–0.24‰, *F_D_* = 0.03–0.7‰) and increases the window of device optimization, in which the highest 94.5% can be obtained (Figure 5e). The average path obtained by each iteration in the optimization interval, with the shortest average distance of 1.6946 km, is smaller than that of traditional HNN (Figure 5f). Compared to other memristive devices, SOT‐MT devices have higher energy efficiency and optimization precision (**Table** **2**). Hence, the developed SOT‐MTs offer advantages for the design and integration of functionally specific devices and arrays. Through integration with CMOS processes, these devices can be miniaturized, enabling efficient identification and optimized combinations, thus holding promise for improved efficiency.

**Table 2:** Comparison of typical approaches of in‐memory HNN tasks implementation.

References	Device	Write speed/bit	Energy consumption/bit	Energy consumption of array	Synapse number	Model	Implementation range	Success probability
[59]	NbO_x_‐based Mott memristors	0.6 µs	<100 fJ	–	4 Kb	Chaos fluctuation	Fluctuation amplitude 0–6 V	Apply to 8‐city TSP
[40]	HfO_2_‐based RRAM	Direct current	5 nJ (t = 10 µs)	327.68 µJ	4 Kb	Gaussian noise fluctuation	Fluctuation 0–6%	26% for 8‐city TSP
[38]	TaO_x_‐based memristor	2 µs	1.25 nJ	–	10 Kb	Chaotic simulated annealing	Self‐feedback weight from 0.12 to 0.05	90% for 10‐city TSP
[60]	HfO_2_‐based RRAM	13.18 ns	33 fJ	38.92 nJ	10 Kb	Simulated annealing fluctuation	Simulated annealing fluctuation from ≈28% to ≈7%	Apply to 10‐city TSP
[61]	FinFET	–	–	460 nJ	7.3 Mb	Temporal noise fluctuation	Fluctuation ≈4–46%	Apply to 150‐city TSP
Our work	CoFeB‐based magnetic texture	0.5 ns	31.25 fJ	2.05 nJ	4 Kb	Neuromorphic LTP and STP	Mix decaying fluctuation from 0‰ to 1.2‰	94.5% for 8‐city TSP

Though the initial conductance modulation in this prototype is limited (0.28%), this value aligns with operational conductance intervals (0.4–2.8%) in established resistive memory technologies used for in‐memory computing, which exhibit greater drift. System‐level techniques—including weight redundancy, write‐verify schemes, and error correction—can effectively mitigate array‐level variations. Furthermore, significant improvements are projected through material engineering (40% AMR),^[^ ^42^ ^]^ self‐referenced designs doubling read margin,^[^ ^43^ ^]^ and domain‐wall tunnel junctions enabling >100% MR ratios,^[^ ^44^ ^]^ collectively enhancing signal integrity for scalable implementations.

Nevertheless, MNIST and TSP‐8 serve as foundational benchmarks here, their use aligns with established practice in early‐stage neuromorphic device research^[^ ^3^, ^13^ ^]^ where comparative analysis within controlled frameworks is prioritized. These standardized tasks enable direct assessment of computational fidelity against known baselines under device‐specific constraints. We emphasize that these results demonstrate operational principles rather than ultimate performance limits. Future work will scale to more complex problems as device integration matures, with the current benchmarks serving to validate the core physics and functionality essential for neuromorphic implementation.

## 3 Discussion

A neuromorphic system with capabilities for diverse computational tasks offers the potential to enable in‐memory computing and draw inspiration from neuroscience.^[^ ^45^ ^]^ However, designing a hardware system that can accommodate the requirements of both CNN and HNN poses challenges.^[^ ^46^ ^]^ As the complexity of image recognition and combinatorial optimization increases, the hardware architecture must be scalable to accommodate the growing computational demands. In our present study, the complex MT produced by the prototypical Ta/Co_20_Fe_60_B_20_/MgO/Ta heterostructure at room temperature provides an appropriate platform to implement all‐electrically controlled driving and reading. The ubiquitous presence of pinning, oscillation, and creep of the magnetic domains with dynamic fluctuations was originally considered to be adverse effects in practical applications,^[^ ^13^, ^27^ ^]^ leading to an inevitable detrimental influence on deterministic computing. Therefore, it is crucial to innovate an alternative scientific and technological strategy to utilize the capabilities of dynamic fluctuation for the development of CMOS‐compatible multifunctional neuromorphic hardware. Our findings indicate that variations in the oscillation amplitude of MTs, acting as artificial synapses, do not affect the recognition outcomes during performing inference tasks at room temperature within the acceptable range for MNIST recognition. Furthermore, we explored the inherent and intrinsic characteristics of “variation” by utilizing the HNN to address combinatorial optimization problems. We observed that the intrinsic fluctuations exhibited by the device contribute to the attainment of the global optimum point during the memory‐based optimization task of the neural network. Following the application of actual current excitation, Joule heating causes an increase in device temperature, resulting in a gradual reduction in dissipation. This reduction is advantageous for the convergence of the system's energy function during iterations, ultimately stabilizing at the global optimum point.

Given the limitations of the existing material systems in terms of the values of AMR, it is reasonable to expect significant advancements through future research in material and device engineering. On one hand, it is worthwhile to explore the potential novel functional characteristics of MT devices with higher MR ratios by leveraging the existing properties of materials, e.g., La_1.2_Sr_1.8_Mn_2_O_7_ (AMR = 80%),^[^ ^47^ ^]^ Sr_2_IrO_4_ (AMR = 160%).^[^ ^48^ ^]^ On the other hand, at the device level, gating and interface optimization engineering^[^ ^49^ ^]^ to improve SOT efficiency and regulate DMI strength, device miniaturization, and even all‐electronically controlled conversion between stripe domains and skyrmions, can further improve the energy efficiency and multi‐tasking compatibility of devices. Notably, 3D stacking structures^[^ ^16^ ^]^ and more exotic materials and textures are expected to offer the fascinating prospect of in‐materio computation based on complex physical effects.

The present magnetoresistance ratio, while sufficient to demonstrate the core concept, impacts the energy efficiency of the readout circuitry in a crossbar array. The energy dissipation of the analog‐to‐digital converters (ADCs), a dominant energy cost, scales exponentially with the required bit precision.^[^ ^50^ ^]^ To achieve favorable system‐level energy dissipation, a considerable on‐off ratio is required, as this relaxes the ADC precision requirements, enabling the use of low‐power, low‐resolution converters.^[^ ^51^ ^]^ We anticipate reaching this target through material engineering, utilizing compounds with colossal AMR,^[^ ^48^ ^]^ and by evolving the device architecture from a planar Hall bar to a perpendicular magnetic tunnel junction (MTJ). Domain‐wall‐based MTJs have been shown to function as high‐endurance analog memristors with large read‐out signals, perfectly aligning with the needs of our neuromorphic synapse.^[^ ^39^ ^]^

The scalability of our SOT‐MT devices is governed by the material parameters defining magnetic texture stability—PMA, exchange stiffness, and DMI.^[^ ^18^ ^]^ As dimensions shrink, enhanced interface effects can promote denser textures. Our simulations and prior work on nanoscale spin textures^[^ ^52^, ^53^ ^]^ indicate that functional labyrinthine domains can be maintained down to ≈200 nm. Beyond this, a transition to single‐domain or skyrmionic states presents a viable pathway for miniaturization, as such topological states also offer dynamic properties suitable for neuromorphic computing.^[^ ^54^ ^]^

To address temperature stability in practical implementations, we propose several on‐chip strategies. The primary solution employs a differential readout architecture where each active SOT‐MT synapse is paired (as elaborated in Figure 5) with a reference device of identical structure but with a fixed magnetic state. This configuration effectively cancels anomalous common‐mode temperature drift while preserving the AMR signal. Supplementary approaches include integrated temperature sensing for dynamic compensation, and pulsed readout schemes synchronized with the device's short‐term relaxation characteristics (Figure 4b) to minimize drift impact.

Note that the dynamics of our SOT‐MT devices open up exciting pathways for implementing more complex neural networks beyond the multi‐layer perceptron and Hopfield network demonstrated here. The device's short‐term plasticity and temporal response make it a natural hardware primitive for Recurrent Neural Networks (RNNs),^[^ ^55^, ^56^ ^]^ potentially enabling efficient processing of sequential data like time series or language by leveraging the device's own physics as a memory element. Furthermore, for Generative Adversarial Networks (GANs),^[^ ^57^, ^58^ ^]^ the intrinsic conductance fluctuations of the device array could serve as an on‐chip, analog noise source for the generator's input, simplifying system architecture. Realizing these advanced networks will be the focus of future work as we progress toward larger‐scale integrated systems.

## 4 Conclusion

In summary, we have presented a novel neuromorphic hardware device based on a Ta/Co_20_Fe_60_B_20_/MgO heterostructure with stable complex magnetic topologies at room temperature. The device can be fully controlled electrically and shows potential for in‐memory neural networks, enabling multitasking capabilities for advanced computing tasks. Leveraging the multi‐resistance regulation and observed characteristics of LTP and STP, we successfully modulated synaptic weights and achieved convergence to stable conductance values. Our device outperformed existing neuromorphic devices in recognition tasks and solving the TSP. The complex MTs enhanced read response, and future advancements in energy efficiency, miniaturization, and read window can be anticipated. This paves the way for spin‐in‐memory neuromorphic edge computing systems and brain‐like neural connections. Integrating visual recognition with combinatorial optimization will lead to more intelligent, efficient, and automated decision‐making across various domains, contributing to the development of more capable cognitive computing systems.

## 5 Experimental Section

Specific details regarding device fabrications, electrical measurements, magnetic texture dynamics visualization, micromagnetic simulation, the implementation of handwritten digits recognition, and the traveling salesman problem can be found in the Supporting Information.

## Conflict of Interest

The authors declare no conflict of interest.

## Author Contributions

Y.Z., Y.L., and H.L. contributed equally to this work. G.Z.X. conceived the core idea of this study. Y.F.Z., H.L., G.Z.X., Y.L., D.W., L.L., and Z.H.Z. performed the experiments and measurements. Y.F.Z., H.L, G.Z.X., D.W., and L.L. performed the micromagnetic and magneto‐resistance simulations. G.Z.X., Y.F.Z., Y.L., H.L., X.Y.W., D.W., L.L., G.L.X., Z.H.Z., Y.S., and T.W. performed the analysis. G.Z.X., H.L., Y.F.Z., and Y.L. prepared the manuscript. All authors contributed to the interpretation of the results and discussions.

## Supporting information



Supporting Information

Supplemental Video 1

Supplemental Video 2

Supplemental Video 3

## Data Availability

The data that support the findings of this study are available from the corresponding author upon reasonable request.
