# Supplementary material for: Harnessing Time‐Dependent Magnetic Texture Dynamics via Spin‐Orbit Torque for Physics‐Enhanced Neuromorphic Computing
Source: Adv Sci (Weinh). 2025 Nov 10;13(12):e13946. doi: 10.1002/advs.202513946 (PMC12948257; doi:10.1002/advs.202513946)
Supplement: Supplementary file 1 — Supporting Information [file ADVS-13-e13946-s003.docx]

Supporting Information

Harnessing time-dependent magnetic texture dynamics via spin-orbit torque for physics-enhanced neuromorphic computing

*Yifan Zhang, Yu Li, Huai Lin, Xinying Wang, Long Liu, Guoliang Xing, Di Wang, Zhihao Zhao, Zhipeng Guo, Junbo Yang, Jiebin Niu, Yan Sun, Tom Wu and Guozhong Xing**

Y. F. Zhang, Y. Li, L. Liu, D. Wang, Z. Z. Zhao, Z. P. Guo, J. B. Yang, J. B. Niu and G. Z. Xing

State Key Laboratory of Fabrication Technologies for Integrated Circuits, Institute of Microelectronics, Chinese Academy of Sciences, Beijing, 100029, China

University of Chinese Academy of Sciences, Beijing, 100049, China

***E-mail: (gzxing[@ime.ac.cn](mailto:zhangfeng_ime@ime.ac.cn))

H. Lin

State Key Laboratory of Fabrication Technologies for Integrated Circuits, Institute of Microelectronics, Chinese Academy of Sciences, Beijing, 100029, China

VeriSilicon Technology (Shanghai) Co., Ltd., Shanghai 201306, China

X. Y. Wang

School of Civil Engineering and Architecture, Northeast Electric Power University, Jilin, 132012, China

G. L. Xing

Jilin Special Equipment Inspection and Research Institute, Jilin, 132013, China

Y. Sun

Shenyang National Laboratory for Materials Science, Institute of Metal Research, Chinese Academy of Sciences, Shenyang, 110016, China

T. Wu

Department of Applied Physics, The Hong Kong Polytechnic University, Hong Kong, China

Yifan Zhang, Yu Li, Huai Lin contributed equally to this work.

# Scheme 1. Experimental and simulation workflow.

# Materials and Methods: Device fabrications, electrical measurements, magnetic texture dynamics visualization, micromagnetic simulation, the implementation of handwritten digits recognition and traveling salesman problem.

**Figure S1.** Films stack device structure with cross-sectional high-angle annular dark-field scanning transmission electron microscopy (HAADF-STEM) image, key elements EDS mappings and distribution profiles.

**Figure S2.** Characteristics of developed Ta/CoFeB/MgO/Ta films with different CoFeB thickness.

**Figure S3.** Determination of critical current density (*J*_c_) and Dzyaloshinskii–Moriya interaction effective field (*H*_DMI_).

**Figure S4.** Simulated magnetic domain motion evolution and magnetoresistance response driven by independent current.

**Figure S5.** Resistance measurement results of synaptic SOT-MT devices.

**Note S1.** Elaboration on temperature stability and proposed readout schemes.

**Table S1.** MuMax^3^ simulation parameters.

**Video S1.** Magnetic texture transforms from initial random morphology to parallel orientation, which is consistent with the experiments carried out.

**Video S2.** Overall morphology of magnetic texture remains randomness and inconspicuous motivation with sequence STT current pluses.

**Video S3.** Dynamic magnetic texture evolution from disorderly to orderly arrangement driven by utilizing SOT pulses.

**Scheme 1.** Experimental and simulation workflow.

# Materials and Methods:

**Device fabrications**

On the conventional substrate Si/SiO_2_, the optimized conditions were used to grow Ta(2nm)/Co_20_Fe_60_B_20_(t~nm)/MgO(1.2nm)/Ta(3nm) multilayer samples by magnetron sputtering (t=1.1~1.5nm), further study growth conditions such as Co_20_Fe_60_B_20_ growth thickness, and sputtering power were explored to improve anisotropy and current drive efficiency. Among them, Ta and Co_20_Fe_60_B_20_ materials were grown with a DC power supply, the growth pressure is about 2 mTorr, the power is about 15 W, the growth rate is about 0.05 Å/s for Ta, and about 0.077 Å/s for CoFeB; the specific composition ratio of Co_20_Fe_60_B_20_ was used to achieve a better interface contact and anisotropic modulation. The MgO layer was grown with a radio frequency power source, the growth pressure was about 1 mTorr, the power was about 100 W, and the growth rate was about 0.1 Å/s. The metal and oxide multilayer films are grown sequentially by DC and RF power sources, and the growth conditions were further optimized by characterizing magnetic properties, and finally high-quality magnetic multilayer films are obtained. A photolithography machine with a resolution of 1 μm (Zeiss MA6) was utilized to expose micro-nano magnetic domain devices, combined with a dedicated argon ion etching system to fabricate the magnetic texture devices. Finally, the top of the through-hole was covered with Ti(20 nm)/Au(80 nm) electrodes. This multistep fabrication process enabled the precise and controlled manufacturing of micro-nano magnetic domain devices with tailored magnetic textures and well-defined electrical contacts.

**Electrical measurements**

For device read and write operations, the Keithley 6221 current source was employed to deliver current pulses. The readout of the device's voltage response and fluctuations was accomplished using the Keithley 2182A voltmeter, with a read current magnitude set at 10 μA (*J*_e_=4.44×10^6^ A/m^2^). This experimental setup enabled the precise control and measurement of electrical signals during the device characterization process.

**Magnetic texture dynamics visualization**

The acquisition of magneto-optical Kerr effect images involves the implementation of a MOKE system, i.e., MagVision, which utilizes a magnetic field aligned parallel to the sample to achieve sample saturation and capture a reference image. Subsequently, the oscillation demagnetization method was employed to eliminate any residual magnetization, while simultaneously inducing a random and intricate magnetic domain structure in the absence of remanence. Following the application of current pulse driving, images were captured, and the initial reference image was subtracted from the acquired images to obtain the final MOKE image. This process enables the visualization and analysis of magnetic domain behavior and dynamics in the studied system.

**Micromagnetic simulation**

The simulation of intricate magnetic domain walls involves the utilization of MuMax^3^, a micromagnetic simulation software. This simulation takes into account the effects of STT originating from the ferromagnetic layer^[1]^ and the SOT resulting from the interplay between the heavy metal layer and the ferromagnetic layer^[2]^ during current-driven processes. These effects are incorporated into the simulation through the utilization of the Landau-Lifshitz-Gilbert-Slonczewski (LLGS) equation, which provides a comprehensive framework for understanding and analyzing the dynamics of magnetic domain walls in complex systems^[3]^：

$\frac{dm}{dt}=-\gamma m\times H_{eff}+\alpha m\times\frac{dm}{dt}+\gamma\tau_{STT}+\gamma\tau_{SOT}$ (3)

where **m** is the normalized magnetic moment, *γ* is the gyromagnetic ratio, *α* is the Gilbert damping coefficient. **H***_eff_* denotes the effective field as:

$H_{eff}=H_{demag}+H_{ex}+H_{ani}+H_{DMI}$ (4)

where, **H**_demag_ is the demagnetization field, **H**_ex_ represents the exchange bias effective field. **H**_ani_ denotes the anisotropic field; **H**_DMI_ presents as the DMI interaction field.

Device dynamics are characterized by experimental resistance measurements showing stochastic fluctuations over a 500 μs operational timescale in the large-scale prototype, corresponding to domain wall reorientation under current drive. Crucially, micromagnetic simulations (SI Movie) of a scaled-down device (1 µm × 1 µm) confirm the fundamental physics enables significantly faster operation: identical current density drives deterministic magnetic ordering perpendicular to current flow within 500 ps. This projected sub-nanosecond switching is competitive with state-of-the-art approaches (e.g., skyrmion-based systems requiring complex field control), while offering distinct advantages: purely electrical control of resistance states eliminates external magnetic fields, enhancing integration potential. The combination of validated physics, scalable speed, and all-electric operation positions this platform as a promising candidate for high-speed neuromorphic implementations upon miniaturization.

**Handwritten digits recognition**

A deep neural network (DNN) algorithm was implemented to perform digital identification using SOT-MT devices on the MNIST database. A comparison was made between the DNN algorithm and an ideal software identification system. The training process was conducted offline using MATLAB, with the neural network structure consisting of 784 input neurons, 100 neurons in the hidden layer, and 10 neurons in the output layer, corresponding to the recognition of 10 Arabic numeral digits. The training was performed over 100 epochs, and a separate test dataset of 10,000 images was used for evaluation. The synaptic weights were normalized and represented with 4-bit precision. The weight map incorporated possible fluctuations of the magnetic devices. Following the training phase, the inference process was carried out, and the accuracy was calculated after a certain number of iterations.

**Travel salesman problem**

A typical AGI application of SOT-MT devices was investigated to optimize the TSP, which is a classical combinatorial optimization problem. Specifically, we focused on evaluating the effect of magnetic domain device fluctuations on the optimization process. To tackle the 8-city TSP problem, we adopted an iterative approach. Initially, a non-optimal path was selected as the initial solution, and this value remained constant throughout subsequent horizontal comparisons. During the iterative process, we aimed to minimize the objective function, represented by the weight matrix. The ideal value of the weight matrix was determined as^[4]^:

$T_{xi,yj}\text{= }-B\delta_{xy}(1-\delta_{ij})-B\delta_{ij}(1-\delta_{xy})-Cd_{xy}(\delta_{j,i-1}+\delta_{j,i+1})$ (5)

where *B* and *C* are constants, the Kronecker delta function *δ*_ij_=1, if *i*=*j*, and 0 in other cases. In the iterative process, the energy function is written as:

$E(v)\text{= }\frac{B}{2}\sum_{x=1}^{n} (\sum_{i=1}^{n} V_{xi}-1)^{2}+\frac{B}{2}\sum_{i=1}^{n} (\sum_{x=1}^{n} V_{xi}-1)^{2}+\frac{C}{2}\sum_{x=1}^{n} \sum_{y=1}^{n} \sum_{i=1}^{n} V_{xi}d_{xy}V_{xi}V_{y,i+1}$ (6)

where *V* is the input value of the network, *d*_xy_ is the distance between cities xy, n=8, it is 8 cities.

During the iterative process, we implemented a normalization procedure for the resistance value and fluctuation value of the MT device. This normalization was achieved by measuring the resistance fluctuation of the device. Specifically, we selected a Gaussian distribution and time-dependent decay within the range of fluctuation values to accurately simulate the fluctuations introduced by iterative readings in real circuits. The entire iterative process was conducted using the MATLAB environment, providing a controlled and reproducible environment for analysis and experimentation.

**Fig. S1. | Films stack device structure**. **a** Cross-sectional high-angle annular dark-field scanning transmission electron microscopy (HAADF-STEM) image with key elements EDS mappings and distribution profiles **b**.

The high-angle annular dark-field (HAADF) image in Figure S1a indicates smooth interfaces and high crystalline quality in the stacks. The epitaxial growth of individual layers with clear interfaces was revealed by high-resolution bright-field scanning transmission electron microscope (STEM). The extensive high-resolution TEM images indicate that a virtually perfect single-crystalline and continuous film stack is obtained with specifically defined thickness. The analysis of element distribution and intermixing at the interfaces in the film stack involved a comprehensive approach. It combined cross-sectional high-angle annular dark-field scanning transmission electron microscopy (HAADF-STEM) image data with elemental mapping using energy-dispersive X-ray spectroscopy (EDS). This allowed for the visualization of constituent elements in the developed specimens by displaying characteristic x-ray intensities in a two-dimensional manner. The combination of these techniques provided valuable insights into the distribution of elements and the extent of intermixing at the interfaces in the film stack.

**Fig. S2. | Characteristics of developed CoFeB-based films stack with different CoFeB thickness. a** The *K-H* loops of film with CFB in 1.1~1.5 nm thickness. The inset shows the schematic diagram of stacked film. **b** The corresponding maze DW images by demagnetizing the film samples. **c** The frequency distributions map after two-dimensional fast Fourier transform (2D-FFT) of the images in **b**. **d** The gray level distributions from low frequency area to high frequency area of 2D-FFT maps in **c**. Inset illustrates the DW sizes of difference CFB thickness films. Vibrating sample magnetometer (VSM) data of different sample with OOP **e** and IP **f** magnetic field sweeps.

We stack the as grown films of Ta (1 nm)/CoFeB (t)/MgO (1.2 nm)/Ta (2 nm) where t=1.1-1.5 nm, as the schematic diagram shown in Figure S2a. The out-of-plane (OOP) magnetic field along the *z*-axis (H_z_) is performed using perpendicular magneto-optical Kerr microscope (p-MOKE) with the Kerr signal captured in the step of 1.6 Oe. The normalized Kerr signals show hysteresis loops with the sweeping of H_z_ field, indicating the perpendicular magnetic anisotropy (PMA) for 1.1-1.4 nm CFB thickness at room temperature. It can be seen that loops have significant slope with the increase of the thickness of the CFB layer. This phenomenon also indicates that a labyrinth-like magnetic texture are generated on the surface of the thin film sample during the sweeping process. Supplementary Figure S2b shows the MOKE images of the magnetic structure on the film surface without an external magnetic field. Next, we used 2D-FFT to transform MOKE images into frequency domain, as shown in Figure S2c. Therefore, we can calculate the magnetic domain size in real space by calculating the pixel spacing and gray level in the frequency domain, and the domain size is scaling with increasing CFB thickness. Supplementary Figures S2e and 2f also show the process of out-of-plane magnetic anisotropy (PMA) gradually transforming into in-plane magnetic anisotropy (IMA) as the CFB thickness increases.

According to previous research, we can find the relationship between magnetic domain size (*d*) as a function of CFB thickness (*t*) and the dipolar length d_0_^[5]^:

$d=\alpha te^{(\frac{\pi d_{0}}{t})}$ (1)

$d_{0}=\frac{\sigma_{DW}}{\mu_{0}M_{s}^{2}}$ (2)

where constant *α* is 0.955 in magnetic texture system, μ_0_ is permeability of vacuum, σ_DW_ is domain wall energy and *M*_s_ is saturation magnetization. Therefore, σ_DW_ can be calculated as 2.46 mJ/m^2^.

In order to further analyze the film characteristics and provide experimental support for subsequent simulation parameters, the DW energy σ_DW_^[5]^:

$\sigma_{DW}=4\sqrt{AK_{\text{eff}}}-\pi D$ (3)

where A is the exchange stiffness constant, *K*_eff_ is the effective anisotropy which experiment value is 8×10^4^ J/m^3^ and *D* is Dzyaloshinskii–Moriya coefficient. We conducted the loop shift experiment in 10 μm×50 μm Hall bar device and supplementary Figure S3c shows the Dzyaloshinskii–Moriya interaction effective field (*H*_DMI_) is 560 Oe^[6]^. We can calculate,

$\left| D \right|=\mu_{0}M_{s}H_{DMI}\Delta$ (4)

where the domain wall width $\Delta=\sqrt{A/K_{eff}}$^[7]^. Therefore, according to equation (3) and (4), and the VSM result of CFB 1.3 nm film (*M*_s_=8×10^5^ A/m^2^), we obtain A=1.5×10^-11^ J/m, *D*=0.61 mJ/m^2^. Supplementary Figure S3a and S3b show the SOT characteristic of magnetic texture device.


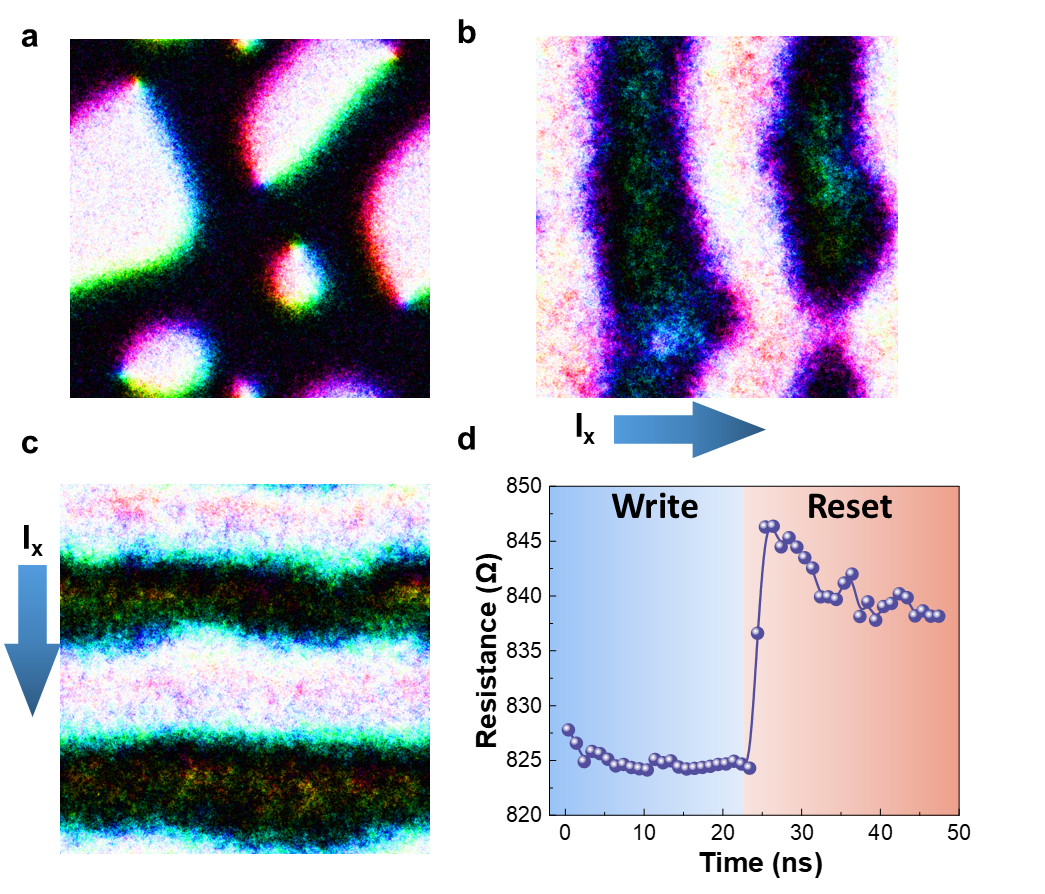


**Fig. S4. | Simulated magnetic domain motion evolution and magnetoresistance response driven by independent current. a** Random magnetic domain structure formed under the initial conditions. **b** A current with a pulse width of 0.5 ns is injected along the *x* direction. Considering the STT and SOT effects, the magnetic domain walls generate an alignment orientation at 16 ns. **c** Change the direction of the current at 22 ns, and inject the current from the *y* direction. The pulse is continuously applied, and the magnetic domains tend to be aligned. **d** Resistance evolution as function of time during write and reset driving operation.

We combine micromagnetic simulation to illustrate the dynamic evolution of magnetic domains and electrical properties during the current driving process. The parameters used in MuMax^3^ are shown in Table 1, where the parameters are derived from experimental results.

Supplementary Figure S4a shows the simulation results show a magnetic bubble structure in the initial state, under room temperature conditions without external fields. The same state can also be seen in the experimental MOKE image in Figure 2c. Then, the magnetic textures tend to be oriented perpendicular to the direction of current flow, after applying 16 current pulses along the *x*-axis, as shown in Figure S4b. Furthermore, we change the current direction to *y*-axis at 22 ns. The orientation of the magnetic textures also gradually changes and are finally perpendicular to the Y-axis direction, as shown in Figure S4c. Finally, we calculate the magnetization matrix^[8]^ during the dynamic process and obtain the dynamic change of the device resistance along the x direction, as shown in Figure S4d. When driven by current in the same direction, the resistance gradually decreases, while the current in the perpendicular direction increases the resistance, which is also consistent with experimental observations. It reflects the modulation effect of pulse current on the magnetic domain morphology and device resistance at the micro level.

While the experimental configuration utilized large-scale devices (~100 µm) with through-hole current injection to resolve complex domain wall dynamics, this approach inherently introduces current non-uniformity unsuitable for direct micromagnetic simulation at that scale. Consequently, our simulations employed a scaled-down model (1 µm × 1 µm) with uniform current density, utilizing the experimentally derived magnetic parameters from SI Table I. This validated that identical current density drives analogous magnetic ordering perpendicular to current flow within 500 ps, demonstrating the operational principle's scalability and the path toward practical neuromorphic implementations with sub-microsecond operation upon miniaturization.

Regarding the scalability of our SOT-MT devices, the fundamental limit is governed by the material parameters that stabilize complex magnetic textures, namely the PMA, the exchange stiffness, and the DMI^[9,10]^. As device dimensions shrink, interface effects become dominant, potentially enhancing PMA and DMI and allowing for denser magnetic textures. Based on our micromagnetic simulations and the observed stabilization of spin textures in nanoscale systems^[11-13]^ we estimate that devices with a critical dimension of ~200 nm can maintain functional labyrinthine domains. Further scaling may transition the device into a single-domain or skyrmionic state, which, as explored in other spintronic neuromorphic systems^[14]^, also possess dynamic properties suitable for brain-inspired computing. This outlines an alternative pathway for the miniaturization of our technology.


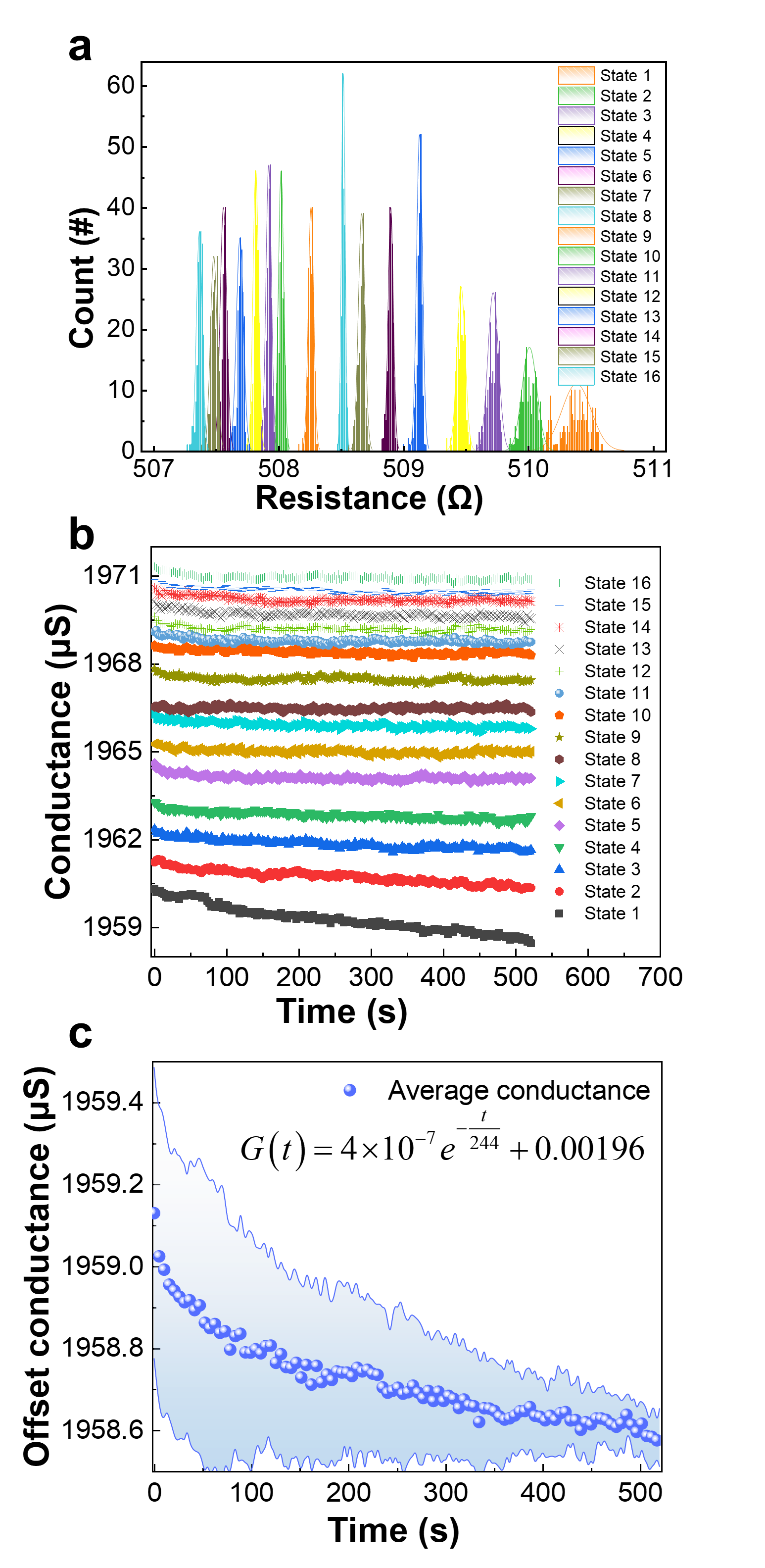


**Fig. S5.** **| Resistance measurement results of synaptic SOT-MT devices.** **a** Distribution of resistance states of device 16. **b** Time-dependent distribution of 16 resistance states. **c** Time-dependent fluctuation modeling.

Supplementary Figure S5a reveals the statistical distribution of each of the 16 resistance states of the device. The overall distribution of each state conforms to the characteristics of Gaussian distribution, and there is an obvious distinction between each resistance state. However, when we analyze the dynamic evolution of each resistance state over time after the write current pulse is injected, we can find that the conductance of each resistance state has a decay process, as shown in Figure S5b. Supplementary Figure S5c extracts the dynamic model of conductance based on the time change data of multi resistance states: $\Delta G_{D}\left( t \right)=\Delta G_{0}exp\left( -t/\tau\right)$. The overall conductance is $G\left( t \right)=4\times10^{-7}e^{-\frac{t}{244}}+0.00196$, which evolves with time and tends to a stable Gaussian fluctuation.

**Note S1.** Elaboration on temperature stability and proposed readout schemes.

To ensure reliable operation of SOT-MT devices in practical applications, we address temperature-induced resistance variations through both material characterization and circuit architecture. Our experimental characterization across 10-350 K reveals that the fundamental temperature-dependent fluctuation (0.015‰ at room temperature) remains approximately an order of magnitude smaller than the operational fluctuation attenuation in each resistance state. This behavior is consistent with the understanding that temperature-dependent scattering in CoFeB exhibits spin-independent characteristics^[15,16]^, where domain wall scattering contributions diminish at elevated temperatures. The substantial ~100:1 ratio between our AMR signal (0.28%) and the temperature-induced baseline fluctuation (0.001-0.003%) provides a favorable foundation for implementing effective compensation strategies.

For array-level implementations, we propose a differential readout architecture that employs reference devices with identical structure to the active SOT-MT synapses but with fixed, pinned magnetic states. During read operations, current passes through both active and reference devices simultaneously. A differential amplifier then measures the voltage difference between them, effectively canceling common-mode signals—including temperature-dependent resistance drift in the Ta layer—while preserving the magnetoresistance signal originating from the dynamic magnetic texture in the free layer. This approach ensures robust operation across temperature variations while maintaining signal integrity, aligning with established precision analog design principles.

Table S1. MuMax^3^ simulation parameters.

| **Parameter** | **Description** | **Value** |
| --- | --- | --- |
| *M_s_* | Saturation Magnetization | 8.0×10^5^ A/m |
| *K_u_* | Uniaxial anisotropy energy | 4.2×10^5^ J/m^3^ |
| *D* | DMI constant | 0.56 mJ/m^2^ |
| *A* | Exchange constant | 1.5×10^-11^ J/m |
| *θ* | Spin Hall angle | -0.2 |
| *V* | FL size | 1 μm × 1 μm × 1.3 nm |
| *T* | Temperature | 300 K |
| *t_W_* | Pulse width | 0.5 ns |
| *J_STT_* | STT current | 2.15×10^11^ A/m^2^ |
| *J_SOT_* | SOT current | 0.392×10^11^ A/m^2^ |

**Supplementary References**

[1] S. Zhang, Z. Li, *Phys. Rev. Lett.* **2004**, *93*, 127204.

[2] L. Liu, O. J. Lee, T. J. Gudmundsen, D. C. Ralph, R. A. Buhrman, *Phys. Rev. Lett.* **2012**, *109*, 096602.

[3] S. Ziętek, J. Mojsiejuk, K. Grochot, S. Łazarski, W. Skowroński, T. Stobiecki, *Phys. Rev. B* **2022**, *106*, 024403.

[4] S. Kumar, J. P. Strachan, R. S. Williams, *Nature* **2017**, *548*, 318.

[5] M. Schott, A. Bernand-Mantel, L. Ranno, S. Pizzini, J. Vogel, H. Béa, C. Baraduc, S. Auffret, G. Gaudin, D. Givord, *Nano Lett.* **2017**, *17*, 3006.

[6] T. Dohi, S. Fukami, H. Ohno, *Phys. Rev. B* **2021**, *103*, 214450.

[7] H. Lin, N. Xu, D. Wang, L. Liu, X. Zhao, Y. Zhou, X. Luo, C. Song, G. Yu, G. Xing, *Advanced Intelligent Systems* **2022**, *4*, 2200028.

[8] D. Prychynenko, M. Sitte, K. Litzius, B. Krüger, G. Bourianoff, M. Kläui, J. Sinova, K. Everschor-Sitte, *Phys. Rev. Applied* **2018**, *9*, 014034.

[9] S. Woo, K. Litzius, B. Krüger, M.-Y. Im, L. Caretta, K. Richter, M. Mann, A. Krone, R. M. Reeve, M. Weigand, P. Agrawal, I. Lemesh, M.-A. Mawass, P. Fischer, M. Kläui, G. S. D. Beach, *Nature Mater* **2016**, *15*, 501.

[10] C. Moreau-Luchaire, C. Moutafis, N. Reyren, J. Sampaio, C. A. F. Vaz, N. Van Horne, K. Bouzehouane, K. Garcia, C. Deranlot, P. Warnicke, P. Wohlhüter, J.-M. George, M. Weigand, J. Raabe, V. Cros, A. Fert, *Nature Nanotech* **2016**, *11*, 444.

[11] J. Xu, L. Xi, S. Xing, J. Sheng, S. Li, L. Wang, X. Kan, T. Ma, Y. Zang, B. Bao, Z. Zhou, M. Yang, Y. Gao, D. Wang, G. Wang, X. Zheng, J. Zhang, H. Du, J. Xu, W. Yin, Y. Zhang, S. Zhou, B. Shen, S. Wang, *ACS Nano* **2024**, *18*, 24515.

[12] M.-G. Han, J. A. Garlow, Y. Liu, H. Zhang, J. Li, D. DiMarzio, M. W. Knight, C. Petrovic, D. Jariwala, Y. Zhu, *Nano Lett.* **2019**, *19*, 7859.

[13] F. Tejo, D. Toneto, S. Oyarzún, J. Hermosilla, C. S. Danna, J. L. Palma, R. B. Da Silva, L. S. Dorneles, J. C. Denardin, *ACS Appl. Mater. Interfaces* **2020**, *12*, 53454.

[14] C. H. Marrows, J. Barker, T. A. Moore, T. Moorsom, *npj Spintronics* **2024**, *2*, 12.

[15] D. Ravelosona, A. Cebollada, F. Briones, C. Diaz-Paniagua, M. A. Hidalgo, F. Batallan, *Phys. Rev. B* **1999**, *59*, 4322.

[16] P. M. Levy, S. Zhang, *Phys. Rev. Lett.* **1997**, *79*, 5110.
